# Did COVID-19 Policies Have the Same Effect on COVID-19 Incidence Among Women and Men? Evidence From Spain and Switzerland

**DOI:** 10.3389/ijph.2022.1604994

**Published:** 2022-09-20

**Authors:** Carmen Sant Fruchtman, Fabienne Beatrice Fischer, Laura Monzón Llamas, Maryam Tavakkoli, Daniel Cobos Muñoz, Marina Antillon

**Affiliations:** ^1^ Swiss Tropical and Public Health Institute (Swiss TPH), Allschwil, Switzerland; ^2^ University of Basel, Basel, Switzerland; ^3^ Independent Researcher, Switzerland

**Keywords:** public health, COVID–19, health policy, epidemiology, gender

## Abstract

**Objective:** This study aimed to investigate how COVID-19 prevention policies influenced the COVID-19 incidence in men and women.

**Methods:** We conducted a retrospective longitudinal study using the Swiss Federal Office of Public Health and the Spanish Ministry of Health surveillance data for February 2020–June 2021 to explore sex and age differences in COVID-19 cases and testing. The female-male incidence rate ratios (IRR) were estimated for each week of the pandemic. We complemented our analysis with qualitative information on relevant containment measures in each country.

**Results:** In Switzerland and in Spain, there was an excess of cases in women of 20–59 years old and 80+. This excess of cases was significant during the waves of the pandemic in both countries. In Switzerland, the biggest difference was observed for the age group 20–29, reaching an excess of 94% of cases compared to men during the first wave of COVID-19 (March–May 2020). The excess of cases in women was greater in Spain than in Switzerland, where it reached 159% for women aged 20–29 during the first wave (March–June 2020). In both countries, the age groups 60–79 had a significant excess of cases in men during the pandemic.

**Conclusion:** COVID-19 public health policies affect men and women in different ways. Our findings highlight the importance of gender-sensitive responses to address a public health crisis.

## Introduction

Early in 2020, the first reports coming from China and Italy indicated the population group with the highest mortality risk due to the virus were men with comorbidities, which was confirmed globally as the pandemic spread [1, 2]. Numerous studies have shown that the impact of the COVID-19 pandemic is gendered: conferring differential risks attributable to both biological differences (sex), but also marked by social dynamics and socially constructed norms (gender) [3]. Given the differences in mortality, much research and academic commentary has focused on explaining the increased mortality in men compared to women [4]. Such approaches, however, have failed to address how sex and gender differences affect COVID-19 incidence [5, 6].

Since early in the pandemic, few countries have routinely reported sex-disaggregated data on cases and deaths of COVID-19 [7]. The countries reporting cases and deaths disaggregated by sex show mostly higher death rates in men and similar incidence for men and women [5]. Unfortunately, incidence data describing the differential progress of the disease by gender is not routinely collected [8]. Gender has been described as a multidimensional variable that describes identity, norms and relations between individuals and that can influence access to health services, social support, as well as behaviour towards the prevention of the virus [9, 10].

A pre-print published in May 2020 examined the apparent equality between men and women in COVID-19 infection rates adding an age-disaggregated analysis in ten European countries [11]. The study used routine epidemiological data and reported a higher rate of infections among women compared to men of working-age (20–59 years old). The difference became non-significant in the population above 60 years of age. This finding illustrates the potential role that social norms could have in the spread of COVID-19.

As the pandemic was unfolding, countries worldwide tried to control its peak with strict public health policies that included lockdowns and other restrictions, which started in March 2020 [12]. Unfortunately, some of these policies reinforced pre-existing inequalities, including gender inequalities [9, 13].

Despite the growing body of evidence showing differences by gender and other social determinants in COVID-19, very few studies have examined how different phases of the pandemic have impacted men and women differentially with a specific focus on COVID-19 incidence and its policy drivers. We explore whether the overall case burden, even in age groups for whom COVID-19 is not usually fatal, shows a similar pattern between men and women and how COVID-19 prevention policies may have affected it.

For this study, we chose to focus on two European countries: Switzerland and Spain, which were among the European countries with the highest numbers of cases and deaths per capita in the first year of the pandemic [14, 15]. Additionally, the countries’ policies represent different stringency to COVID-19 containment approaches, with Spain going into full “lockdown” (as in people were not allowed to leave their homes freely) for a considerable time, while Switzerland never went into full lockdown.

## Methods

We conducted a retrospective longitudinal study using quantitative COVID-19 case and testing data and qualitative data on the containment measures and policies between February 2020 and June 2021 in two European countries, Switzerland and Spain.

### Study Setting

Switzerland is a small federal state in central Europe bordering France, Germany, Austria, the Principality of Liechtenstein and Italy. It is divided into 26 cantons (administrative entities). The Swiss health system is based on universally mandated private health insurance [16].

Spain, bordering Portugal and France and the microstate of Andorra, consists of 17 autonomous communities, including two island territories. It has a public, universally accessible National Health System complemented by voluntary private insurance policies.

Healthcare is more privatised in Switzerland than in Spain, where it is more socialised. Both countries have universal access to healthcare and a decentralised public health system. However, both had mechanisms to centralise decision making in times of an emergency like COVID-19. The per capita spending on health care in 2018 was 9,870 USD in Switzerland, the second highest in the world, while in Spain it was 2,736 USD [17].

Both countries ranked similarly in the latest Gender Gap Index: 10th (Switzerland) and 14th (Spain) place out of 156 [18]. However, despite this high ranking, there are prevailing differences in everyday lives for women and men in these countries. In recent years, men and women have achieved a more balanced participation in the labour market, however, in both countries, most domestic tasks and care work are still predominantly carried out by women in the family context [19, 20]. Even during the lockdown, many women had to consider quitting their jobs to be able to take care of their children, since schools were closed [21].

In both countries, women tend to have jobs that include physical interactions with people (teaching, childcare, health workers, supermarket employees, etc.), many of which were considered “essential”, even when most workers were recommended to stay home [20, 22].

### Data Collection and Analysis

To understand how COVID-19 incidence among men and women changed over time, we used publicly available case data stratified by sex and age. Data was collected from the Swiss Federal Office of Public Health (FOPH), as well as the Spanish Red Nacional de Vigilancia Epidemiológica (RENAVE). COVID-19 cases were mandatory to be notified to the FOPH since before the first case in Switzerland. The case definitions were adapted through time, based on the diagnostic possibilities (changing from PCR-confirmed to rapid test confirmations). Likewise, the definition of a case was updated in Spain according to the technical reports for the COVID-19 case management [23].

We explored the incidence between men and women from the outbreak of the pandemic (February 2020) until June 2021. We calculated the IRR of cases between women and men, stratified by age groups - for each week of the pandemic:

IRRwomen= Cases among women of that age group in that weekPopulation of women in that age groupCases among men of that age group in that weekPopulation of men in that age group

For convenience, excess cases per population are shown in two ways: 1) as IRR, 2) as a percentage deviation from equality between both sexes (IRR = 1):

Percent excess incidence={1IRRwomen−1,IRRwomen<0 (Excess of men)IRRwomen−1,IRRwomen≥0 (Excess of women)

To test for disparities, we used an exact test assuming that incidence is Poisson-distributed. “Waves” in the COVID-19 pandemic were defined as any time that the test positivity rate exceeded 5%, as defined by the World Health Organization (WHO) [24].

We collected information from policies (such as Royal Decree in Spain, or the COVID-19 Ordinance in Switzerland) published by the governments for the regulation of tele-working, school closures, which had been previously hypothesised as the biggest drivers behind gender differences, as well as testing strategies that were regularly updated by the Ministry of Health (MoH) in Spain and the FOPH in Switzerland to complement the case data [25, 26]. Two of the authors (FBF and LM) searched publicly available reports such as official government documents/websites or press releases and press conferences of national-level policies and their implementation at the second administrative level (cantons in Switzerland, autonomous communities in Spain) to create a harmonised timeline of policies in each country. This was an extension of the Health Observatory detailed in a previous manuscript [27]. These data were then visualised to show the duration of policies (for home office recommendations and school closures) and the changes in testing policies.

To account for differences in testing behaviour between men and women, supplemental analyses in Switzerland on testing rates by gender were included and the positivity rate was calculated, stratified by gender. In Spain, testing data stratified by gender was not available.

A simple simulation was performed to calculate the theoretical 95% confidence interval of the positivity rate for women had both genders had the same underlying incidence but women were testing at higher rates. This was done by taking 1,000 Monte Carlo samples from a gamma distribution with shape parameter equal to the cases among men and rate parameter equal to the population of men. Then we sampled from a Poisson distribution with rate parameter equal to the gamma distribution draw times the number of women in the population, and we calculated the simulated positivity-rate by the number of tests done on women in the population.

## Results

### Switzerland

#### COVID-19 Policies Over Time

Switzerland has aimed to strike a balance between limiting the spread of COVID-19 and “normalcy” in social and economic life. A full lockdown, where leaving the house was legally restricted, has never been implemented. The most stringent measures were issued during the first wave from March to June 2020, which included a closure of schools, shops and all leisure and entertainment facilities [25]. Schools were closed from the week of 16 March to the week of 4 May 2020 and most of the remaining restrictions were lifted during summer 2020.

The second wave, which peaked at the end of October 2020, yielded more than seven times as many reported cases but had fewer restrictions and a more diverse response. At this stage, decision making was decentralised to the cantons, which contrasted with the first wave where centralization to the Federal Council was enabled after declaring an “extraordinary situation” as stated in the Epidemics Act [28].

During the second wave, schools remained open, with the exception of universities and other institutions of tertiary education. The second wave was accompanied by a semi-shutdown in which restaurants and other institutions for social activities remained closed for three months. Employers were mandated to enable home office for their employees, if possible. Despite these interventions, the case numbers decreased only slowly between January and March 2021.

During the first months of the pandemic, testing capacities were limited to high-risk groups or people with severe symptoms (Supplementary Figure S1) [29]. Over time and with the availability of more tests, these recommendations became more relaxed and all symptomatic people or people with suspected exposures were included in the testing strategy. From June 2020 onwards, the government would pay for tests if indicated by their testing criteria [30]. By the end of 2020, a rapid antigen test became available and from January 2021, the government agreed to pay for the tests also of asymptomatic people with suspected exposure [31].

Starting in March 2020, the Federal Council implemented a number of social support measures to lessen the impact of the pandemic on companies and employees, such as compensation of loss of earnings for childcare or quarantine/isolation or short time work compensation.

#### Incidence by Sex Over Time

In Switzerland, there were distinct peaks of increasing COVID-19 incidence (“waves”): the first wave in February–April 2020, and the second wave began in October 2020, peaking by the end of the month, and remaining at an overall high level until a third wave in 22 March, 2021.

**FIGURE 1:**
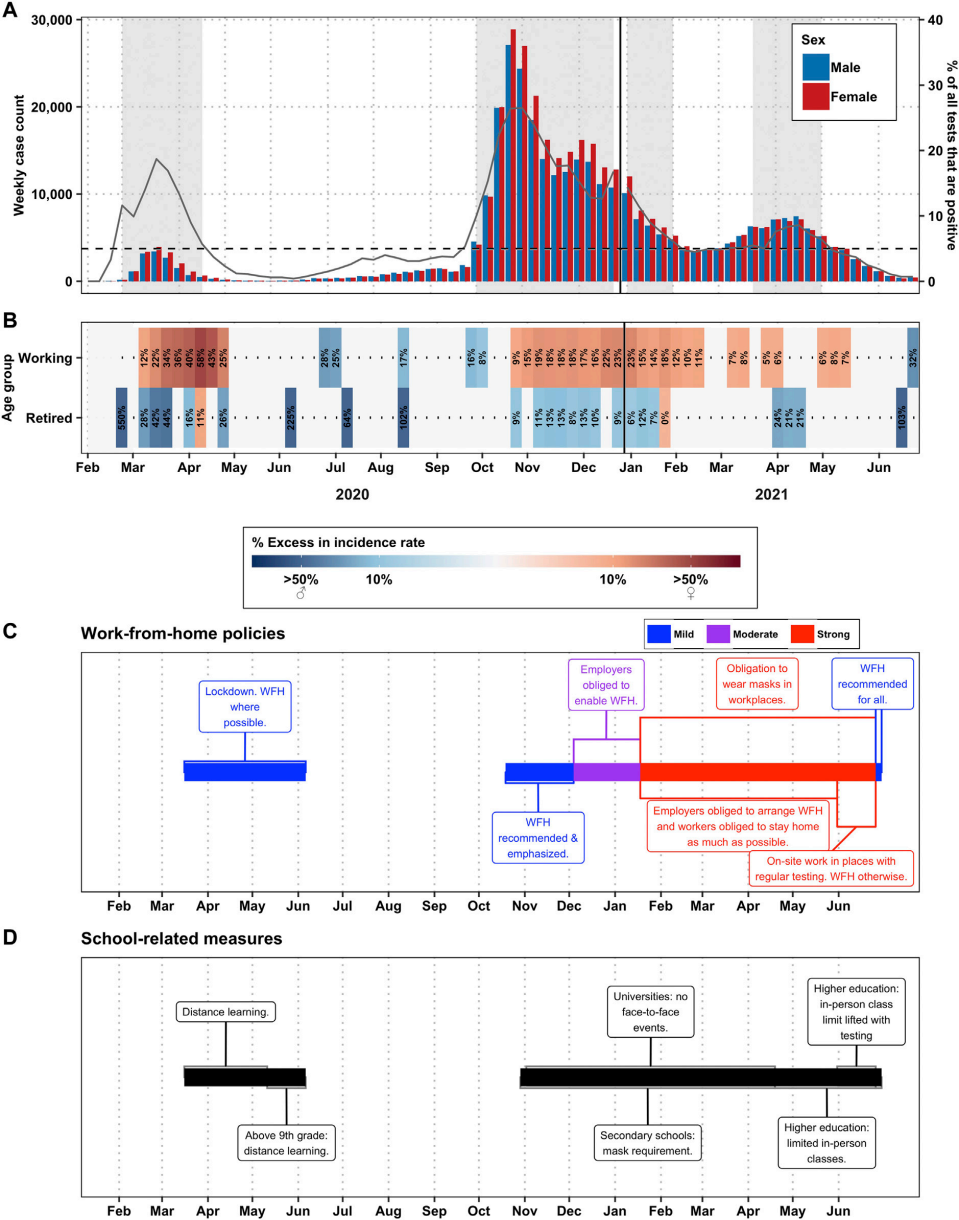
Disparities by gender, in Switzerland (2020–2021)—**(A)** Total number of cases per sex per week. Weeks with backgrounds in grey are weeks where the positivity rate was >5%, the definition of a “wave” in this paper. The gray lines correspond to the % of tests that were positive for that week (right-axis). **(B)** Percent excess in incidence among men (in blue) or among women (in red) by week and by age group. The working age group constitutes ages 20–59 and the retired age group constitutes ages 60–79. People over age 80 were excluded. Weeks marked in white did not have statistically significant differences in the incidence rate ratio between the sexes. **(C)** Work from home policies, color-coded for stringency. **(D)** School policies.

We found that during the waves, women in working ages were significantly overrepresented among all COVID-19 cases (Figure 1). During the first wave, women were overrepresented with an excess of up to 58% among women of working age (20–59 years of age). The excess during the second wave remained at a lower level with a maximum of 23% among women of working age. In contrast, between the waves, little significant difference between the sexes was observed. This is largely attributable to lower case numbers but when the disparity was statistically significant, it disadvantaged men more than women. In an analysis stratified by age (Supplementary Figure S2), there was a tendency towards a higher excess in women aged 20–29 years old with 40–94% excess in the first wave, 10–30% in the second wave, yet only 1 week with a significant difference in the third wave. For older working age groups (30–39, 40–49 and 50–59 years old) the excess was milder than in the 20–29 years old age group. This pattern of changing disparity during the course of the pandemic was not observed for the population of retired age, where men were almost consistently overrepresented among the cases and the weeks with strong disparity were more sporadic and not associated with the waves (Figure 1; incidence rate ratios are shown in Supplementary Figure S3). In the more detailed age group analysis (Supplementary Figure S2), we note that among the retired age group (60–79 years of age), those above 80 years old, men show excess cases in the first wave of up to 80%, but in the second wave women show excess incidence of up to 28%.

**FIGURE 2:**
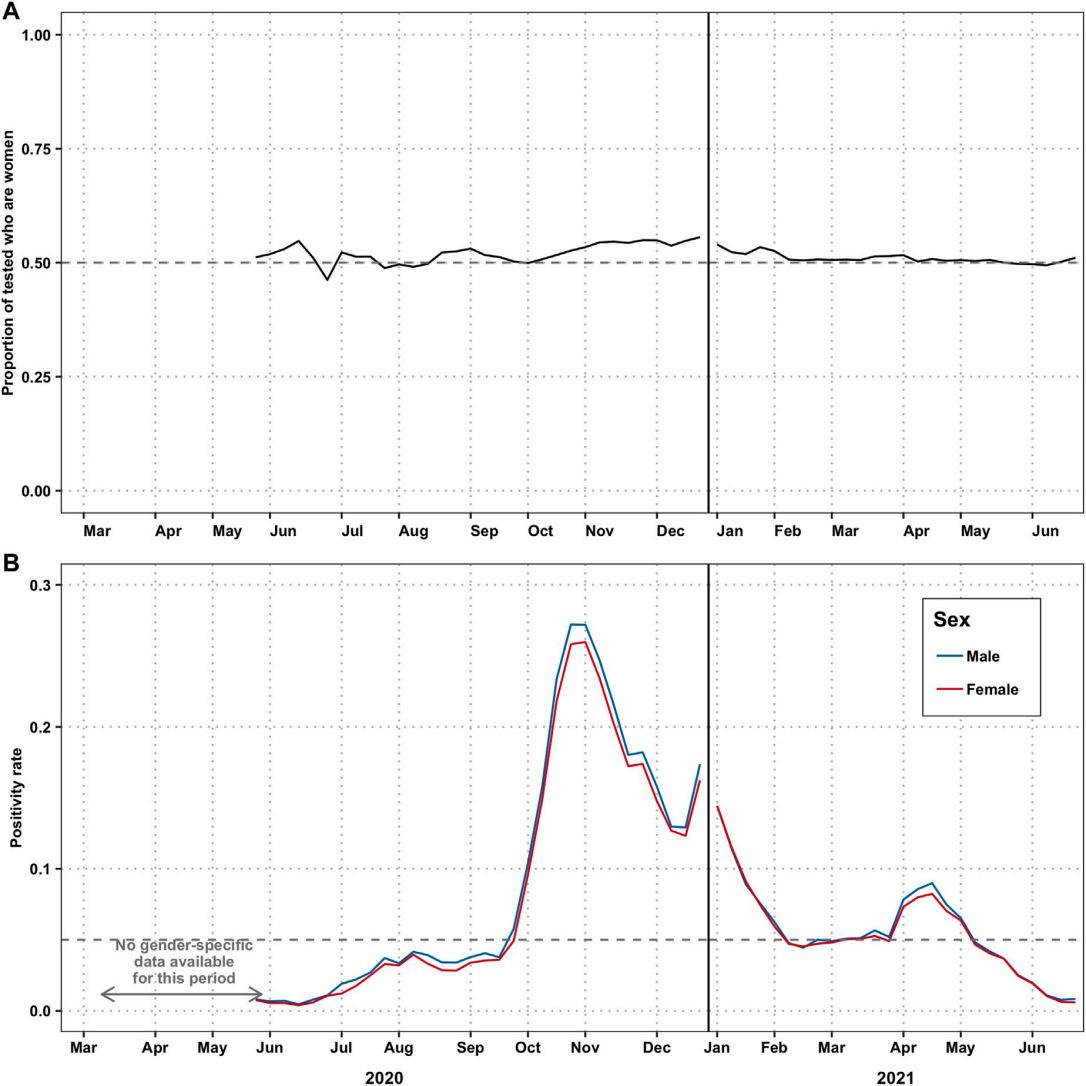
Disparities in testing by gender—(Switzerland. 2020–2021). **(A)** Proportion of tests taken by women. The grey dashed line at 0.5 represents the line at which men and women are testing in equal numbers. **(B)** Proportion of tests among men and women that are positive for COVID-19. We could not perform this analysis from 24 February, when the first case was reported, until the week of 25 May when the positivity rate was first reported stratified by gender. The gray dashed line at 5% represents the WHO-recommended threshold for defining a wave.

In order to assess if our findings were due to testing bias, we analysed testing patterns by sex (Figure 2). The COVID-19 testing rate by sex was only available after the week of 25 May 2020, hence after the first wave. While women were being tested more often than men were, the positivity rate for both men and women was at a comparable level throughout our study period (Figure 2), and the women’s positivity rate was higher than we would expect if the underlying incidence rate was equal to that of the men (Supplementary Figure S4).

### Spain

#### COVID-19 Policies Over Time

**FIGURE 3:**
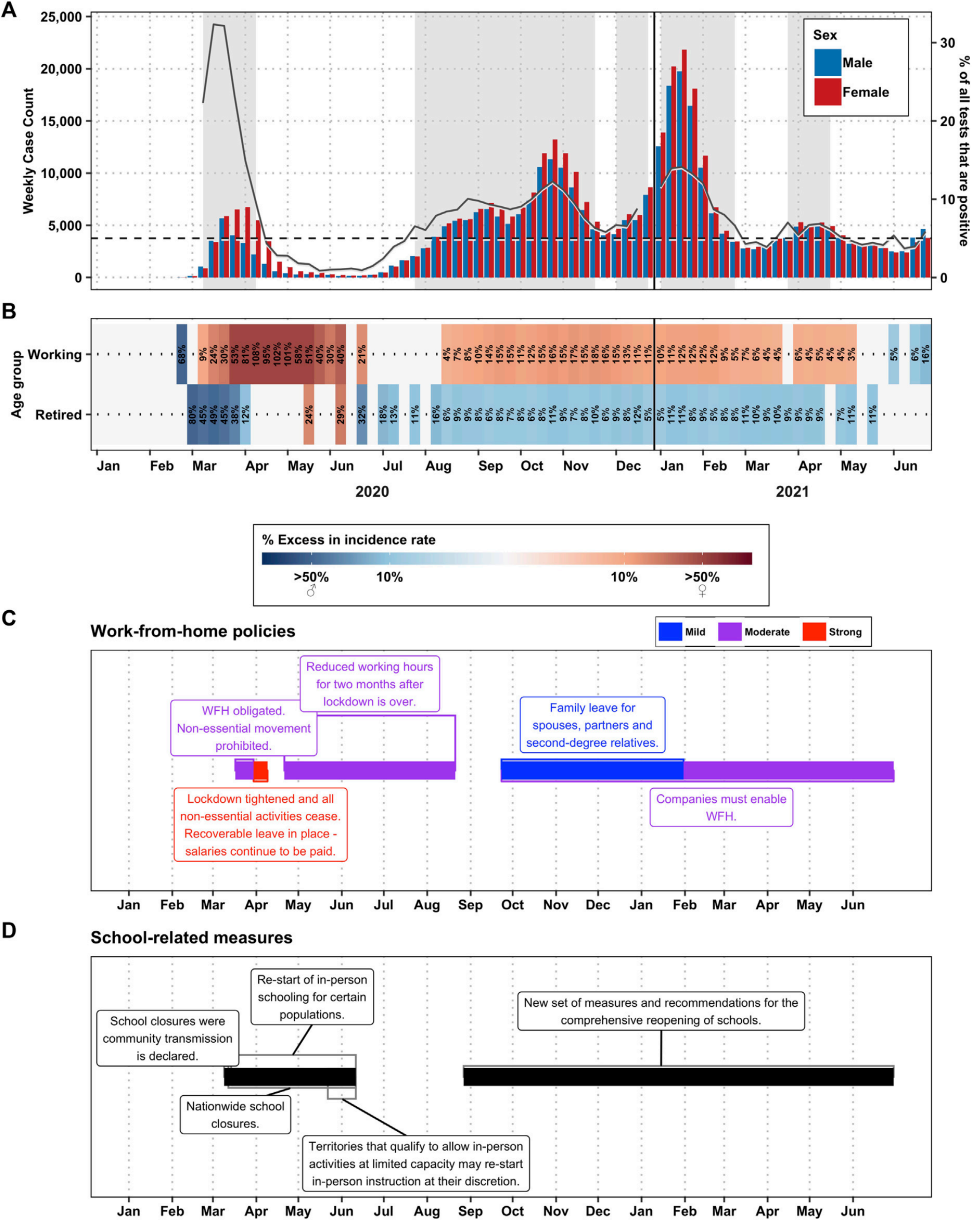
Disparities by gender, in Spain (2020–2021)—**(A)** Total number of cases per sex per week. Weeks with backgrounds in grey are weeks where the positivity rate was >5%, the definition of a “wave” in this paper. The gray lines correspond to the % of tests that were positive for that week (right-axis). **(B)** Percent excess in incidence among men (in blue) or among women (in red) by week and by age group. The working age group constitutes ages 20–59 and the retired age group constitutes ages 60–79. People over age 80 were excluded. Weeks marked in white did not have any statistically significant differences in the incidence rate ratio between the sexes. **(C)** Work from home policies, color-coded for stringency. **(D)** School policies.

In Spain, after the announcement of community transmission, several policy measures were put in place to contain the epidemic (Figure 3). Spain was one of the countries with the most stringent measurements during the first wave. Between March and April 2020 a full lockdown was implemented. Only workers in specific sectors (such as healthcare or retail sector) that were considered essential were allowed to leave their houses. From 9 March, face-to-face education was suspended until September 2020.

Starting on the week of 16 March, teleworking was generally recommended. Between the weeks of 30 March and 6 April, with a total lockdown, all non-essential activities ceased. At that time, health professionals were the most exposed to COVID-19 [32]. After this date, although home office was recommended, the law did not force companies to facilitate it, leaving this decision entirely up to the employer.

COVID-19 testing was implemented nationwide from 13 March (Supplementary Figure S4). However, until 7 May testing was only limited to severe cases of COVID-19 presenting at the emergency department or admitted to the hospital. Health professionals and workers in essential services were also classified as priority populations for testing. Patients with mild and moderate symptoms who were monitored at home or residents in nursing homes were not tested and thus not counted in official statistics of confirmed cases [33]. A study suggested that the lack of tests of non-hospitalized patients could lead to underreporting of cases in women [34]. After 4 May, PCR tests became available for all suspected cases.

Since 28 April 2020, the public health restrictions were slowly lifted and the responsibilities fully devolved to the autonomous communities in a co-governance system. Several waves have been reported since summer 2020: a second wave in October 2020, a third wave in January 2021, and a fourth wave after an intense vaccination campaign between January and June 2021.

#### Incidence by Sex Over Time

Our results show that the most significant gender disparity in relation to COVID-19 incidence was during the first wave in working age groups, reaching an excess of female cases of up to 108% (Figure 3; incidence rate ratios are shown in Supplementary Figure S6). In the detailed age group analysis, we note that the excess is even more pronounced in the 20–29 and 30–39 years-old age groups (Supplementary Figure S5). The excess of female incidence remained during the following peaks, albeit at a lower level, reaching a maximum excess of 18%. In contrast, in the retired age group (60–79 years of age) data shows an excess of male case incidence (reaching 87% of excess) before the week of 6 April 2020. However, this trend changes dramatically in the 80+ age group, where, between the weeks of 6 April through 15 June, the excess of female cases reached 102% (Supplementary Figure S5). After the first wave, there is no significant gender disparity among the retired age groups (less than 12% excess of male cases).

## Discussion

Gender differences in relation to COVID-19 incidence rates have been previously discussed, given the higher risk of mortality and hospitalisation in men [35]. However, less attention has been given to the sex-differential impact of public health response on COVID-19 case incidence rates. The COVID-19 burden goes beyond mortality and short-term illness [36]. Women have been shown to be four times as likely than men to suffer from at least one persisting symptom after a COVID-19 infection for an extended period [37].

Global data suggested a similar case burden in women and men during the first year of the pandemic [38]. Our study shows, however, that in Spain and in Switzerland, during the waves in 2020 and the first half of 2021, more women were diagnosed with COVID-19 than men. Testing data from Switzerland suggests that this phenomenon is not due to differential test-seeking behaviour between the genders, but rather different incidence (Figure 2), as the positivity rate was similar in both groups. Yet, higher infection rates in women are only present for the populations of working age (20–59 years old) and above 80 years old (Supplementary Figure S2). In a previous study, Sobotka et al. also found a higher rate of cases in women compared to men, for the working age group [11].

The difference in the stringency of the containment measures between the waves studied seemed to be associated with a different degree of disparity, which could be read similar to a “dose response” relationship. The more stringent the measures, the larger the gender disparity, which could explain the differences seen between Switzerland and Spain; in Switzerland, where the measures were less stringent, the gender disparity in cases was lower than in Spain.

The WHO’s sex and gender in infectious diseases framework [39] describes the interaction of sex and gender with infectious diseases at three different levels: 1) vulnerability to the disease, 2) ability to prevent exposure and 3) decision-making power.

It has been hypothesised before that women are more exposed than men to COVID-19, be it in the domestic or professional setting [40]. Paradoxically, women also self-reported higher compliance with containment measures (namely social distancing and hygiene) [41]. Therefore, even though women aim to act responsibly, they are limited in their ability to prevent exposure. They are subject to more frequent or more precarious exposures than men, which would explain what we observed in Switzerland and in Spain. We discuss below the potential causal pathways between the implementation of COVID-19 prevention policies and the differential protective effect in men and women.

### Women Were More Exposed to COVID-19 at Work

In Switzerland, when home office policies were established (recommended from 13 March–6 June, 2020, 19 October–4 December 2020, and starting on 26 June 2021; but moderately or strongly advised from 4 December 2020–26 June 2021) the excess of cases among women increased significantly. However, when we looked at the degree of stringency and the degree of excess cases among women, it suggests that it is the school closure during the first wave that is associated with the excess among women, whereas in the more severe second wave, schools remained open for younger children, and the excess among women was milder (Figure 1). A similar situation was observed in Spain, where an excess of cases was observed among the women of working age, and more marked among women of typically child-bearing age during the closure of schools and day care centres (Figure 3).

During the first wave the stringency of policies differed in Switzerland and Spain: while in Switzerland a “soft-lockdown” was applied, citizens in Spain were forced to stay at home. Face-to-face education at schools and universities were suspended in both countries. Exceptions to the norms of staying and working from home were issued in Switzerland as well as in Spain for workers in sectors considered “essential”. Essential services included those ensuring supply of food and hygiene products, medicines, health care, transport or security [27, 42]. Workers in these sectors are predominantly women in both countries [22, 42, 43]. For context, in Switzerland 68% of the health workforce, 92% of childcare and 67% of retail positions are staffed by women [22]. Similarly, in Spain, more than 70% of the health professionals are women and they are also overrepresented in sectors like social work, retail, health and cleaning services [42]. Studies have shown that workers in some industries, such as meat factories, were predominantly male and at higher risk of contracting COVID-19 due to superspreading events [44]. However, in the case of Spain and Switzerland, these occupations account for a smaller volume of workers than the health, educational and care sectors, where women are overrepresented.

There is little to no evidence in both countries on case burden by sex and occupation. However, several studies have shown a higher case burden among the health workforce. Furthermore, Perez-Romero et al. found that most health and social care professionals in several high incidence areas in Spain were infected in their workplaces, while the general population were infected mostly at home [45]. One study in Switzerland found increased seroprevalence in hospitals treating COVID-19 patients compared to hospitals without COVID-19 patients, but overall only a small difference between healthcare workers and the general population was observed [46].

### Care-Giving in Switzerland and Spain

Evidence suggests that women were not only more exposed at work, they were also more exposed to the virus at home compared to men. In Switzerland, women take on more of the unpaid care work than men (31.2% of women reported to take care of either children, adults or both compared to 11.6% of men) [47]. In both countries, if a family member gets ill, it has been shown that the closest care (with the highest infection risks) falls to the women of the household [48]. Additionally, most (known) transmissions in Switzerland happened within households [46].

The closure of schools and nursery homes implied that two out of three mothers had to stay at home in Spain, shouldering the highest burden of domestic and care work [21]. Moreover, the additional burden on women due to caregiving activities did not only increase their risk to contract the virus, but it also led to additional secondary effects, such as loss of jobs.

### Over-Representation of Women in Nursing Homes

Finally, our results also show that women above 80 years old were at higher risk of contracting the disease than men in the same age group. The difference was, however, more prominent in Spain (Supplementary Figures S2 vs. S6), where the difference was maintained throughout the pandemic, reaching 102% of infections in April 2020. The Spanish MoH estimates that almost 20 thousand people died between January and June 2020 in nursing homes nationwide due to COVID-19, where most of the residents are women [21]. Several studies and media reports addressed the problem of nursing homes during the COVID-19 pandemic, as elderly people were abandoned by the State, leaving especially old women in a vulnerable situation [49].

Switzerland could have faced similar challenges, as there was an excess of COVID-19 cases in women above 80 years old during the second wave of the pandemic (November 2020–January 2021). According to the Swiss Federal Statistical Office, in 2020, 1.8% of the Swiss population lived in care homes (either short-term or long-term), and among those living in care homes 67% are women [50]. In a recently published report, an increase of 80% of deaths in care homes was reported during autumn of 2020 [51]. The press release does not differentiate, however, between deaths of men and women. Our study findings, which show an excess of cases in women over age 80 in the second wave (Supplementary Figure S2), highlight the importance of understanding if this increase of deaths was attributable to transmission within care homes.

We hypothesize that the reason for the overrepresentation of women in old-age nursing homes is partly an overall decline in the proportion of men with increasing age, but also that women of that age have often lost their partners, while men could potentially benefit from at-home care from their wives or partners [21, 52]. This circumstance could be attributed to the fact that men tend to have younger partners and a lower life expectancy.

The COVID-19 crisis has affected everyone, but in different ways. Social determinants and inequalities have been described as key factors behind the drivers of this pandemic. Social determinants have influenced the risk of contracting the disease, the outcomes of it, as well as the unintended effects of the containment measures [52]. Our findings for two exemplary countries, Spain and Switzerland, suggest that the differences in the sex-ratio of cases are not only due to biological differences, but rather to social and gender norms and how policies affected population groups differently. These associations were seen despite both of our selected countries ranking high in the Global Gender Gap report; it is likely that our findings are transferable to many other countries.

### Limitations

Our results are probably showing an underrepresentation of disparities, given changing testing policies. Until May 2020, the testing strategy only covered inpatients and severe cases, which were borne in a slightly higher proportion by elderly men. Furthermore, data on testing disaggregated by both age and sex were not available for either country.

### Conclusion

Our study shows that while the mortality of COVID-19 is disadvantageous to men, the incidence of COVID-19 disproportionately burdens women, in particular women of child-bearing and working age (20–59 years old). This has long-term implications due to fourfold higher odds of developing “long COVID” borne by women.

Evidence is emerging about the protective benefits and effectiveness of certain policies and non-pharmaceutical interventions to reduce COVID-19 incidence. These studies are, however, often looking at overall numbers and may overlook how policies may reduce the risk differently among population groups, including those defined by gender. When different effects are observed this is often attributed to the levels of compliance, rather than structural exposures or risks that are unaddressed by the policies. We argue that there is a need to search for drivers beyond compliance and understand how policies enable certain groups to shield from the pandemic more than others.

Policy and decision-makers have embedded gender in their discourse, but this has often been limited to rhetoric or implementing policies to alleviate socioeconomic effects of the pandemic. Our study highlights that a gender perspective is also crucial to implement incidence-prevention measures, like non-pharmaceutical interventions.

## Supplementary Material

Supplementary Figure S1 Testing policies in Switzerland. Dates are listed along the x-axis, and vertical lines indicate the date when the policy was implemented. RDT tests were available in Switzerland since 18 December 2020, but only for screening purposes. Confirmation of a case was only possible through PCR (Switzerland. 2020-2021).

Supplementary Figure S2 Disparities by gender, detailed (Switzerland. 2020-2021). **(A)** Total number of cases per sex per week. Weeks with backgrounds in gray are weeks where the positivity rate was >5%, the definition of a “wave” in this paper. **(B)** Percent excess in incidence among men (in blue) or among women (in red) by week and by detailed age group. Weeks marked in white did not have any statistically significant differences in the incidence rate ratio between the sexes.

Supplementary Figure S3 Incidence rate ratio for COVID-19 cases among women compared to men by week from February 2020–June 2021 and stratified for working age groups and retired age groups (Switzerland. 2020-2021). The solid lines represent the weeks for which the IRR is statistically significant (*p* < 0.05) and the shaded lines represent the weeks for which the IRR is not statistically significant.

Supplementary Figure S4 Sensitivity analysis of testing positivity rate in Switzerland if men and women had the same incidence rate but different testing rates. The gray ribbon shows the expected positivity rate for women when men's incidence rates were applied (Switzerland 2020–2021).

Supplementary Figure S5 Testing policies in Spain. Dates are listed along the x-axis, and grey vertical lines indicate the date when the policy was implemented. RDT tests were available in Spain since 7 December 2020, but only for screening purposes. Confirmation of a case was only possible through PCR. (Spain. 2020–2021).

Supplementary Figure S6 Disparities by gender, detailed (Spain. 2020–2021). **(A)** Total number of cases per sex per week. Weeks with backgrounds in gray are weeks where the positivity rate was >5%, the definition of a “wave” in this paper. **(B)** Percent excess in incidence among men (in blue) or among women (in red) by week and by detailed age group. Weeks marked in white did not have any statistically significant differences in the incidence rate ratio between the sexes.

Supplementary Figure S7 Incidence rate ratio for COVID-19 cases among women compared to men by week from February 2020–June 2021 and stratified for working age groups and retired age groups (Spain. 2020–2021). The solid lines represent the weeks for which the IRR is statistically significant (*p* < 0.05) and the shaded lines represent the weeks for which the IRR is not statistically significant.
