# Supplementary figures and images for: Did COVID-19 Policies Have the Same Effect on COVID-19 Incidence Among Women and Men? Evidence From Spain and Switzerland
Source: Int J Public Health. 2022 Sep 20;67:1604994. doi: 10.3389/ijph.2022.1604994 (PMC9530041; doi:10.3389/ijph.2022.1604994)

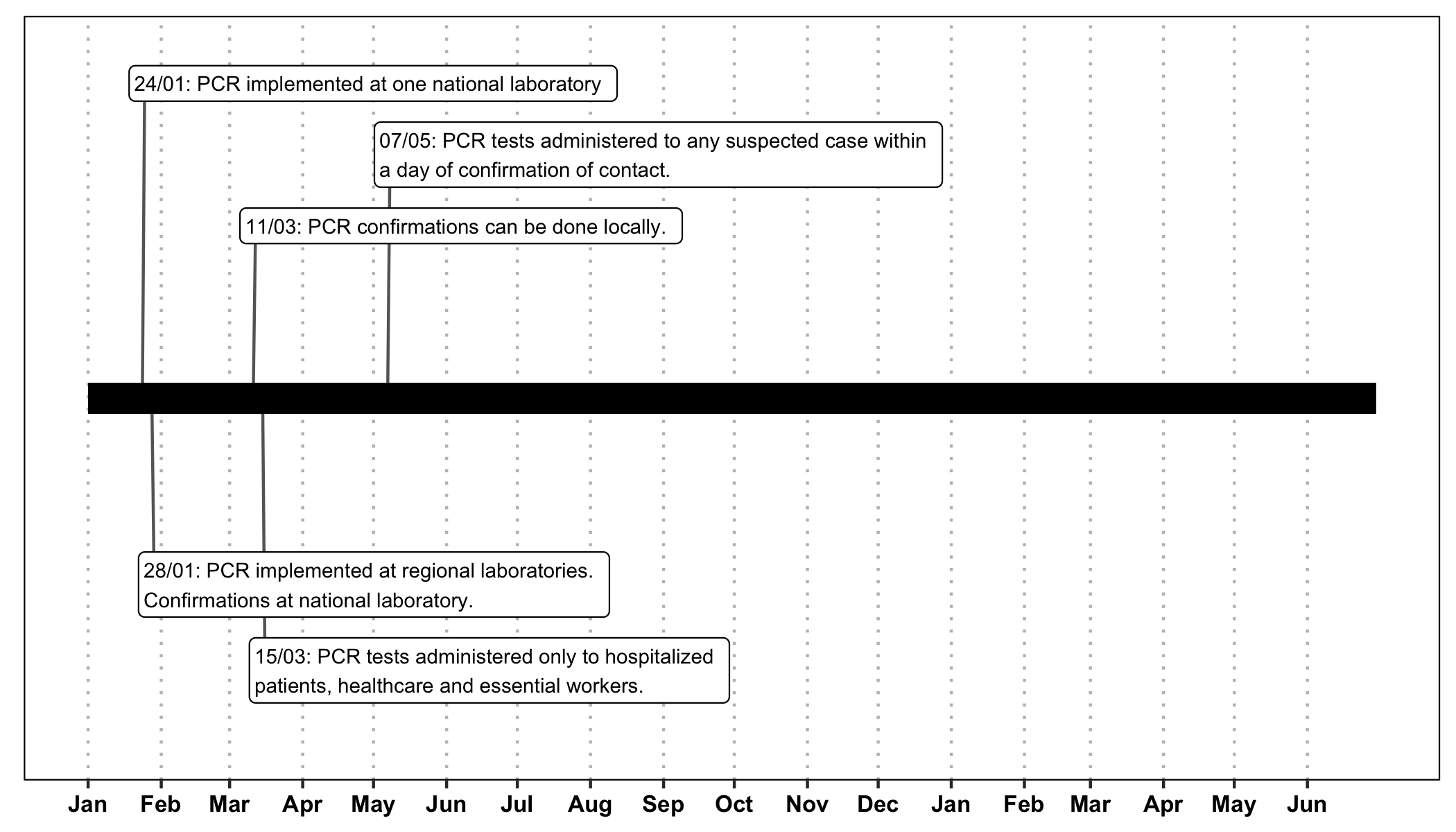

Supplement: Supplementary file 1 [file Image5.PNG]

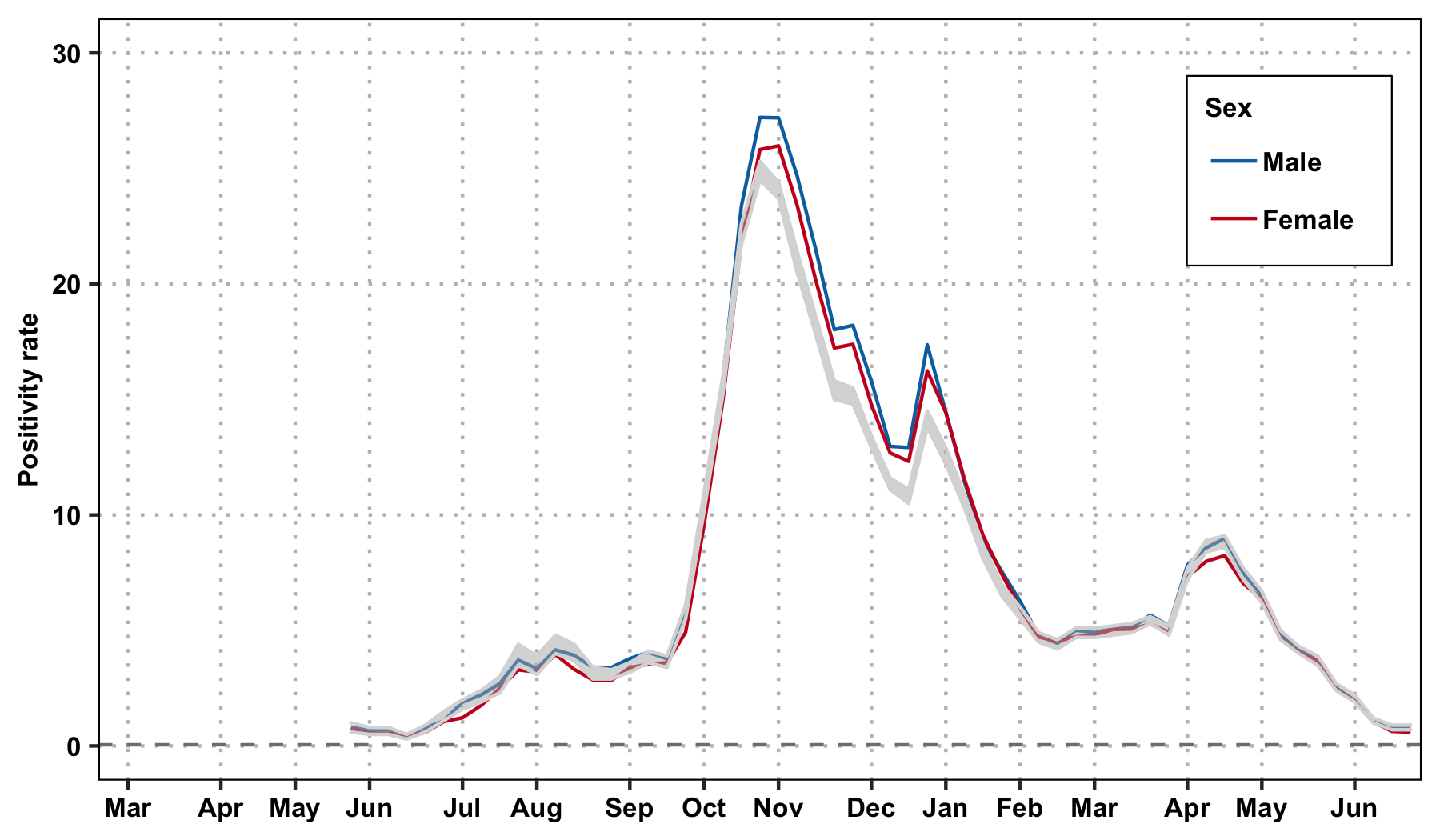

Supplement: Supplementary file 2 [file Image4.PNG]

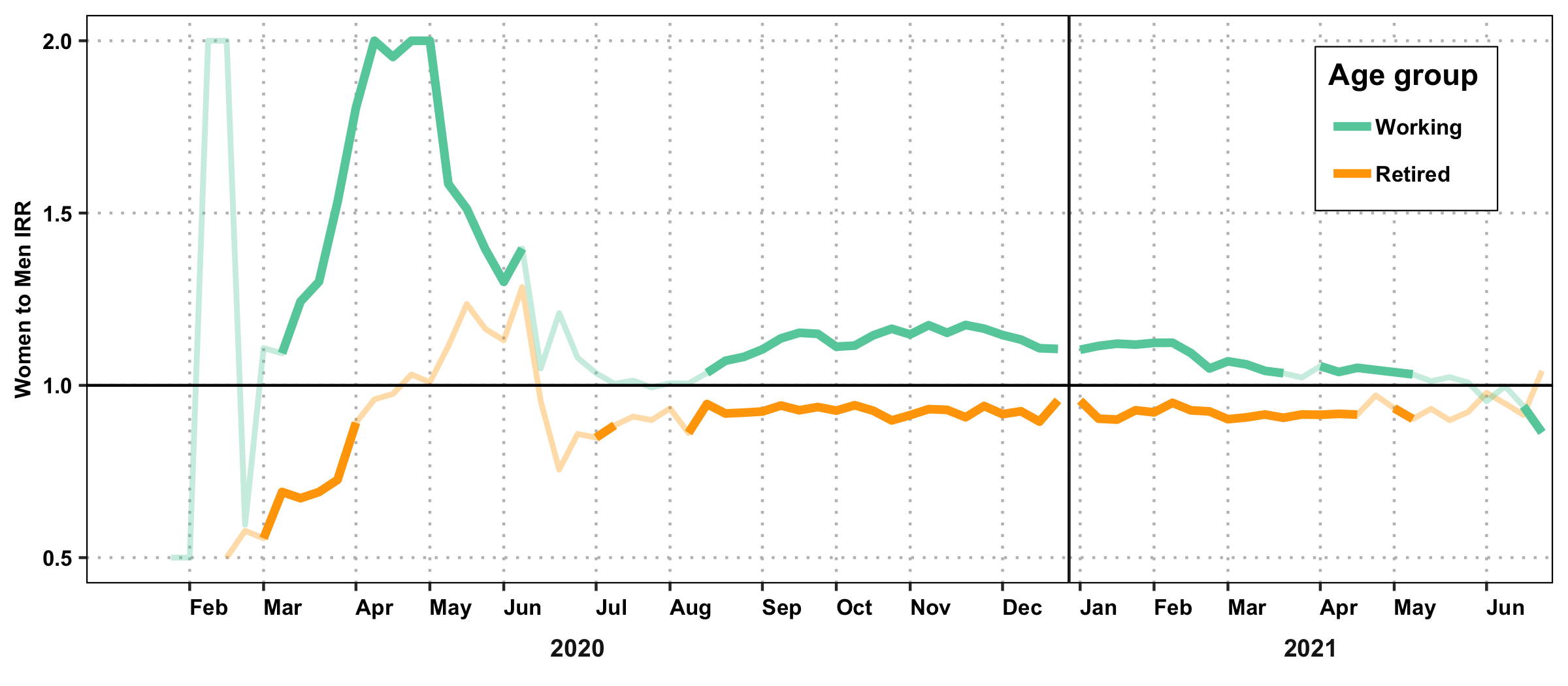

Supplement: Supplementary file 3 [file Image7.png]

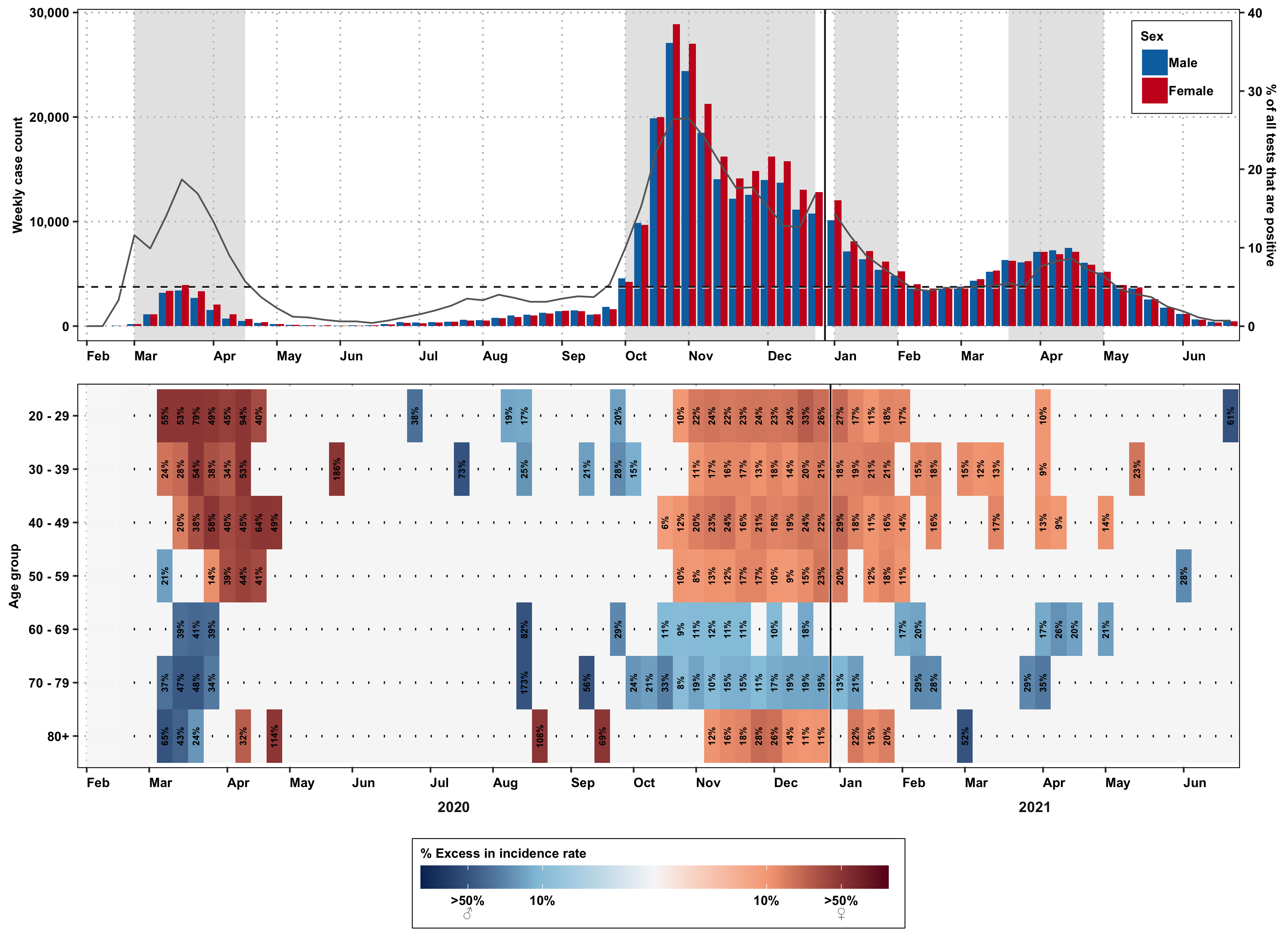

Supplement: Supplementary file 4 [file Image2.PNG]

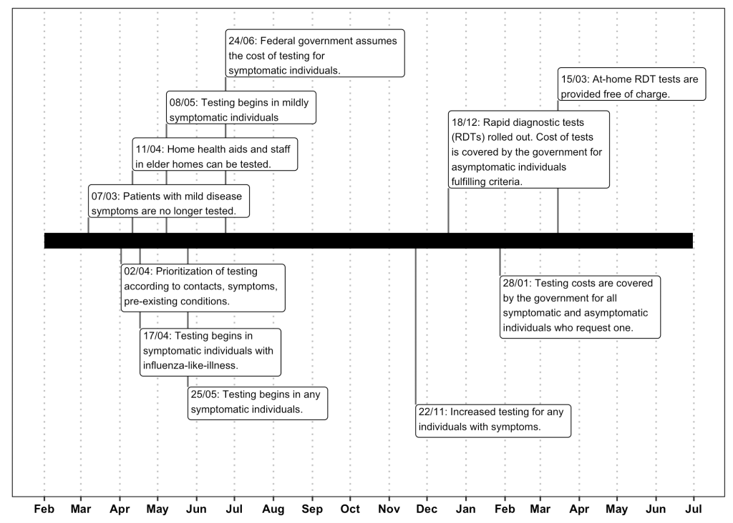

Supplement: Supplementary file 5 [file Image1.PNG]

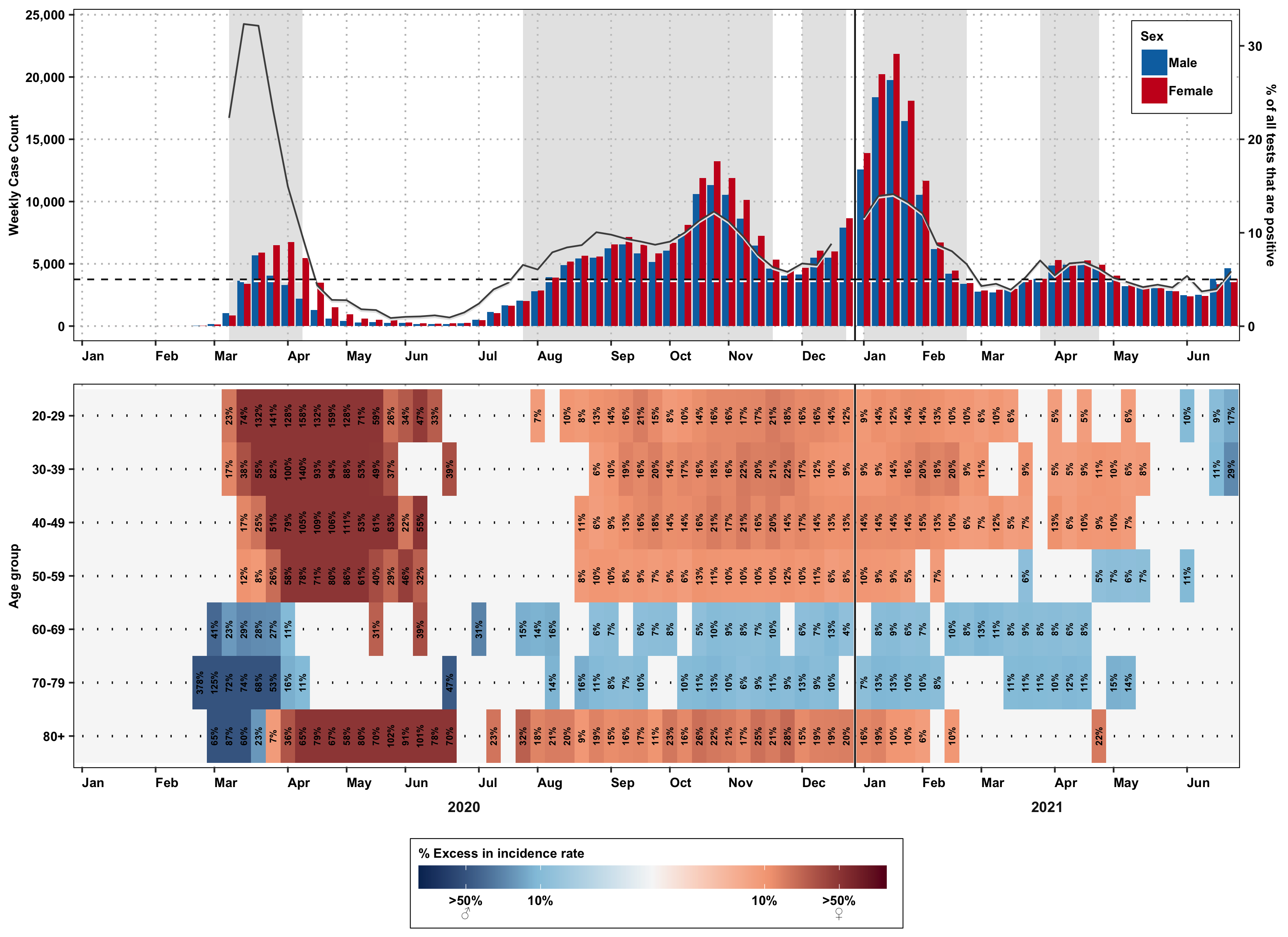

Supplement: Supplementary file 6 [file Image6.PNG]

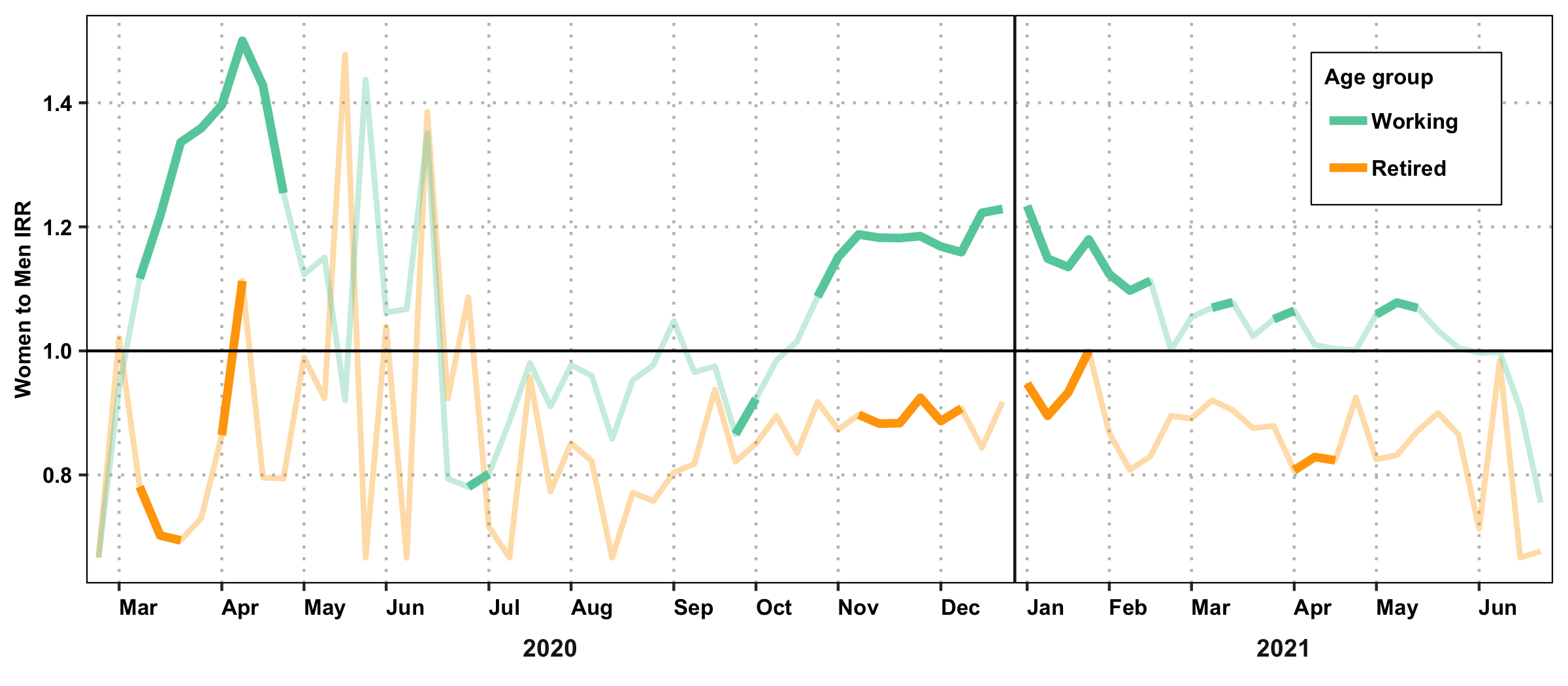

Supplement: Supplementary file 7 [file Image3.PNG]
